# Correction to “Beyond the Michaelis‐Menten: Evaluation of a tQSSA‐Based IVIVE Approach for Predicting In Vivo Intrinsic Clearance from Hepatocyte Assays”

**DOI:** 10.1002/psp4.70216

**Published:** 2026-02-12

N.‐A. T. Vu, Y. M. Song, S. K. Kim, et al., “Beyond the Michaelis–Menten: Evaluation of a tQSSA‐Based IVIVE Approach for Predicting In Vivo Intrinsic Clearance From Hepatocyte Assays,” *CPT: Pharmacometrics & Systems Pharmacology* 15, no. 2 (2026): e70169, https://doi.org/10.1002/psp4.70169.

In the article cited above, the authors inadvertently omitted the Acknowledgments section, which is shown below.

The authors regret this error.

